# Supplementary material for: Morpho-Physiological and Transcriptome Changes in Tomato Anthers of Different Developmental Stages under Drought Stress
Source: Cells. 2021 Jul 17;10(7):1809. doi: 10.3390/cells10071809 (PMC8305550; doi:10.3390/cells10071809)
Supplement: Supplementary file 1 [file cells-10-01809-s001.zip › cells-1291549-supplementary/Suplementary Files/Supplementary Figures.pdf]

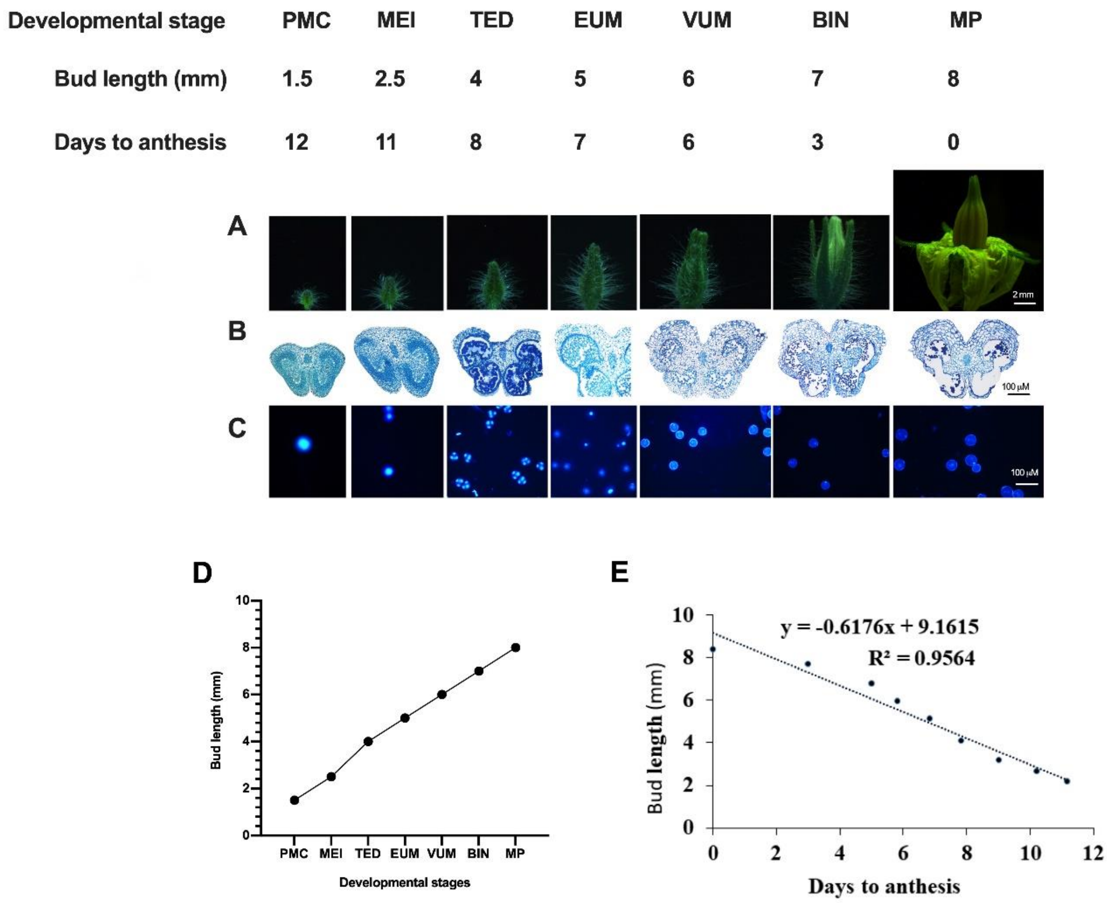

Figure S1. Anther/pollen development in tomato (S.lycopersicum) cultivar Micro-Tom and relationship between floral bud size and duration to anthesis. Anther/pollen development in (*Solanum lycopersicum*) cultivar 'Micro-Tom' Anther/ pollen development was examined at six landmark stages from pollen mother cell to mature stage anthers (**A**) The name, floral bud/flower length (mm) and days to anthesis of specific developmental stages are indicated above A. The development stage and floral bud/flower length were correlated by measuring the length of at least 3 floral buds/flower and conducting histological observations (**B**) and staining with DAPI (**C**). Then the floral bud length and/or stage of development were correlated with time to anthesis by measuring the length and tagging/recording the position of 10 floral buds for each developmental stage from 3 biological replications. Each floral bud was monitored to determine the time (d) it took to anthesis. A direct correction between floral bud length and stage of development was observed (**D**) whereas an inverse relationship between floral bud length and days to anthesis was observed (**E**). PMC, pollen mother cell; MEI, meiotic; TED, tetrad; EUM, early uninucleate microspore; VUM, vacuolated uninucleate microspore, BIN, binucleate; MP mature pollen.

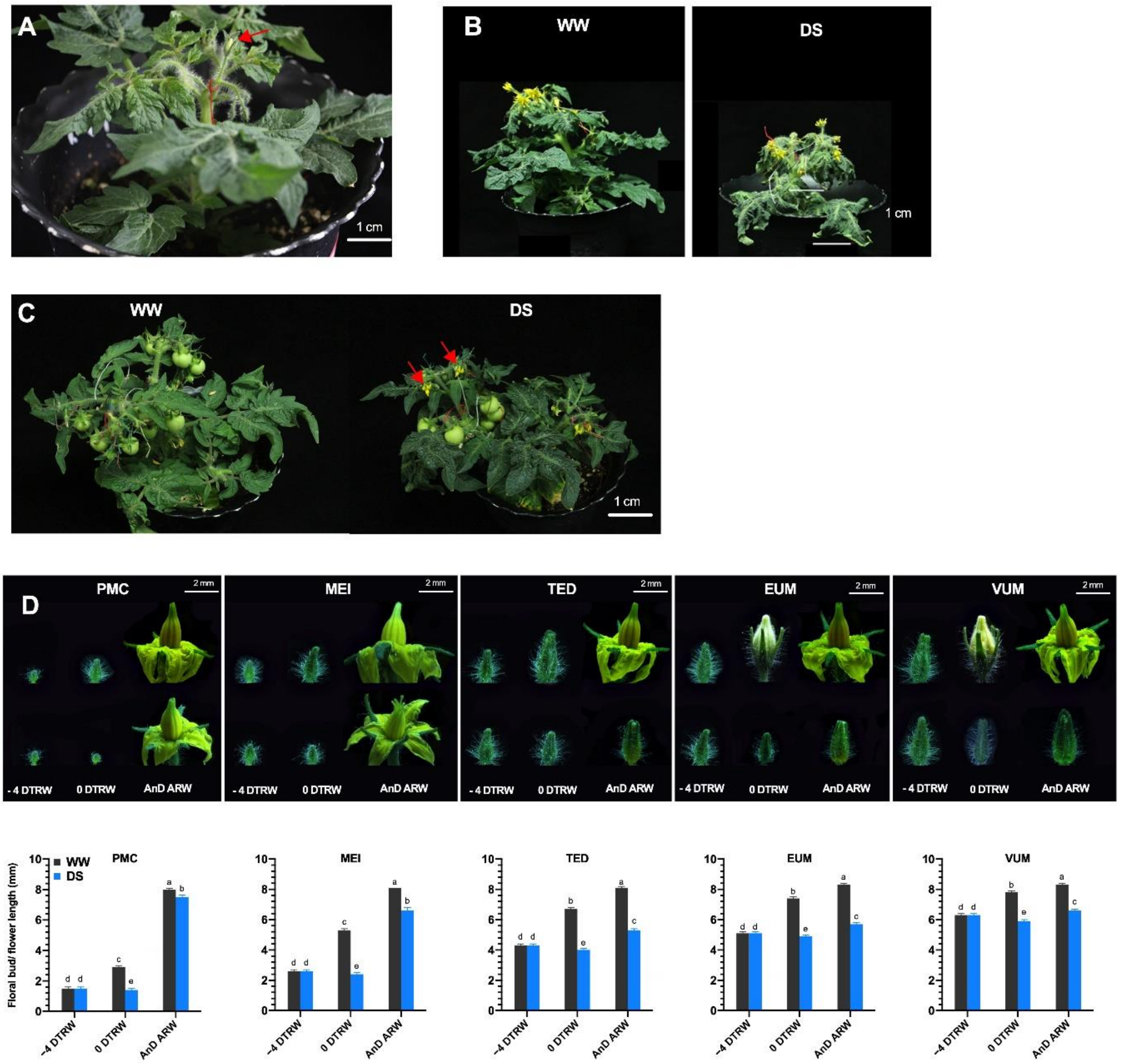

Figure S2. Floral bud development under drought stress and rewatering. (**A**) A tomato plant at reproductive stage, 35 days after sowing with first emerged floral bud at binucleate stage of anther/pollen development (arrow) on the first truss (tagged red) at the time watering was suspended**.** (**B**) Flowering WW and DS tomato plants at 0 DTRW, the end of the 4 days drought stress. The DS plant was clearly stunted. (**C**) WW plant and DS-rewatered plant 11 DARW. Flower produc-tion in the WW plants had ended while DS-rewatered plants continued with the second peak of flower production (ar-rows). (**D**) Drought stress caused reversible and irreversible growth inhibition in tomato anthers at different stages of development. Data presented are means ±SD (*n* = 10). Bars with different letters are significant different (LSD $_{0.05}$).

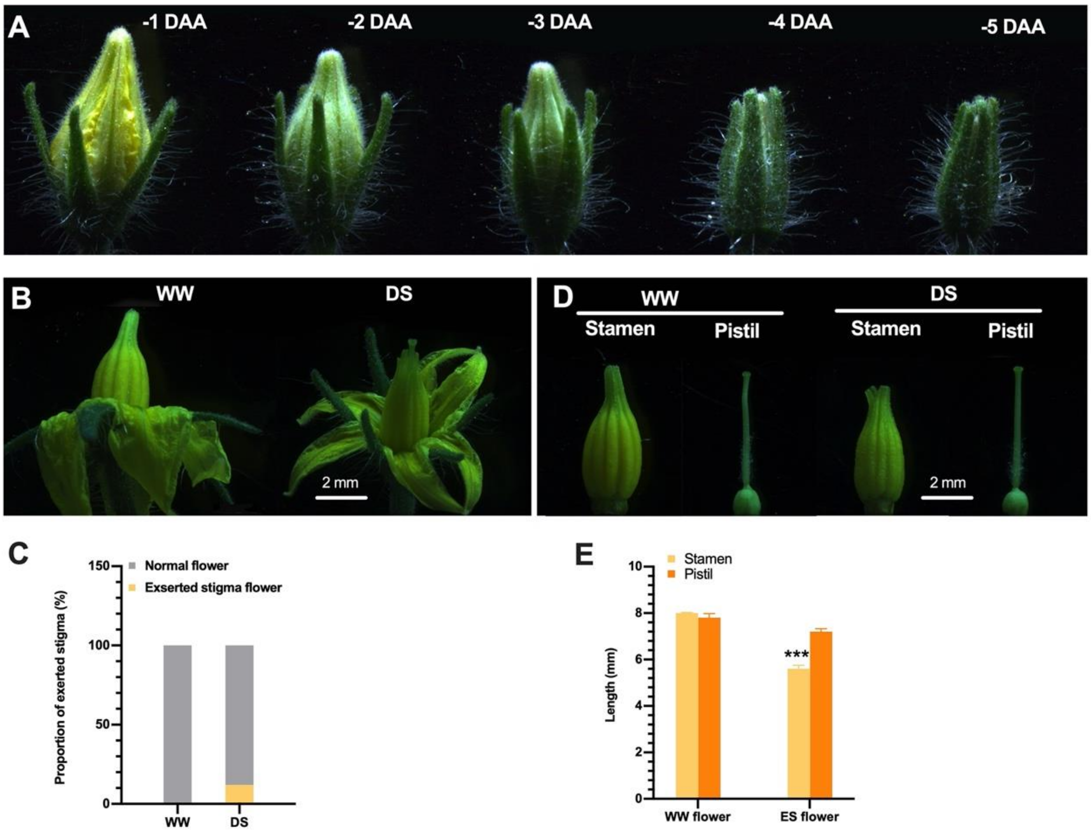

Figure S3. Binucleate to mature (BIN-MP) floral buds at onset drought stress period and stigma exsertion in tomato caused by drought stress. (**A**) Morphology of group 3 floral buds at the start of DS period when soil moisture reached 6% i.e. −4 DTRW. From left to right: binucleate (BIN, −1DAA), BIN (−2DAA), BIN (-3DAA), Early binucleate (EBIN, −4 DAA), tran-sition to pollen mitosis 1(TPM1, −5 DAA). During the DS, the −1, −2 and −3 DAA floral buds reached anthesis during −3,2 and −1 DTRW respectively. At 0 DTRW, the −4 DAA floral bud reached anthesis and the –5 DAA anther remained uno-pened but was at an advanced stage of development i.e. at −1 DAA. DAA, days to anthesis. Often flowers do not open daily on a single truss, therefore the above floral buds were obtained from WW plants for the purpose of demonstration. (**B-E**) Drought stress induced stigma exsertion in flowers drought stressed at Meiotic stage. (**B**) Morphology of normal flower from WW plant and exserted stigma flower from DS plant 9 days after rewatering. (**C**) Proportion of flowers with exserted stigma from all recorded floral buds that reached anthesis in DS plants. (**D,E**) Length of stamen and pistil within exserted stigma flower compared. Values are means ± SD (n>10). *** p <0.0001(t-test). WW, well-watered; DS, drought stress; ES exserted stigma.

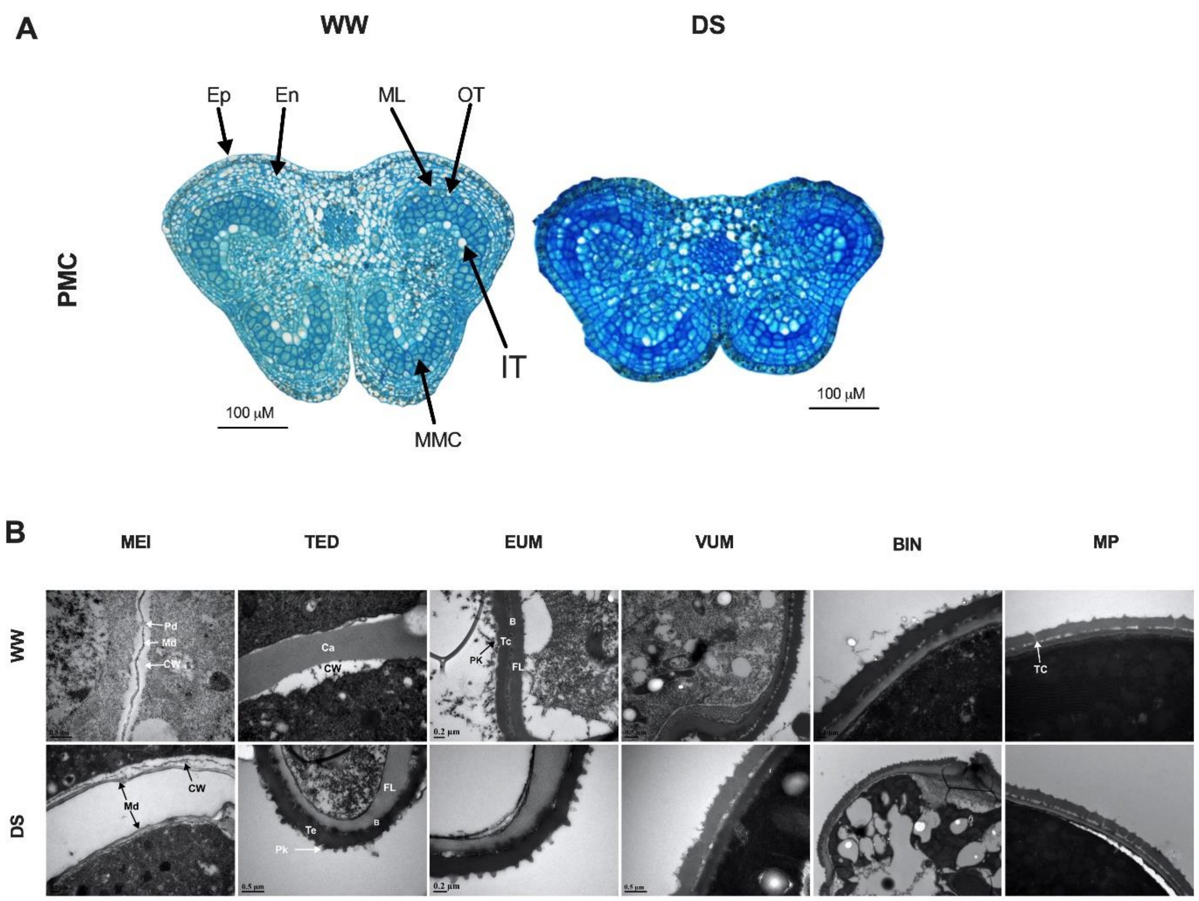

Figure S4. Histology of pollen mother cell anthers and pollen wall development under drought stress. (A) Cross-sections of anthers at pollen mother cell stage from WW and DS plants after 4 d of drought stress. WW, well-watered; DS, drought stressed; Ep, epidermis; En, endothecium; ML, middle layers; OT, outer tapetum; IT, inner tapetum; MMC, microspore mother cell. (**B**) Transmission electron micrographs of developing pollen wall from anthers at six different stages of development under DS. (WW), wall of pollen from anthers of well-watered plants; (DS), wall of pollen from anthers of drought-stressed plants. Pd, plasmodesma; Md, middle lamella; Cw, cell wall; Ca, callose wall; B, baculum; Tc, tectum; FL, foot layer; PK, pollenkit; TC, tectal chamber.

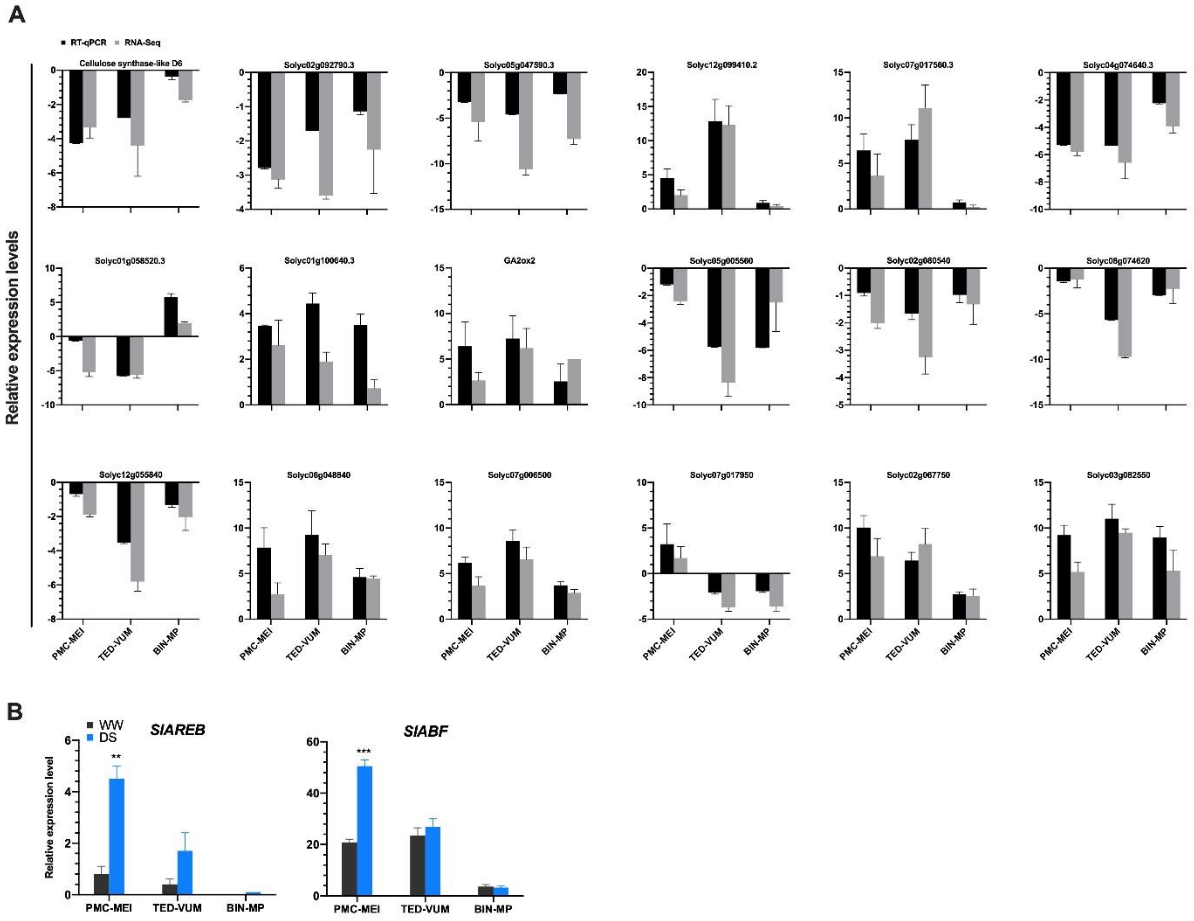

Figure S5. Confirmation of RNA-Seq results by RT-qPCR and expression analysis of transcriptions factors in ABA signal-ling. (**A**) Comparison of RNA-Seq and RT-qPCR results of 18 randomly selected genes. PMC-MEI, pollen mother cell-meiotic stage anthers; TED-VUM, tetrad-vacuolated uninucleate microspore stage anthers; BIN-MP, binucleate-mature pollen stage anthers. Data presented as means ±SD (n = 3 replications, 24 plants). *SlUbi3* was used as the internal control geneduringRT-qPCRanalysis.(**B**) Expression analysis based on RNA-Seq data of TFs genes involved in the regulation of ABA-dependent drought responsive genes expressions. Values are means ±SD (n = 3 replications, 24 plants each) ** $P < 0.01$, *** $P < 0.001$ (T-test).
