# Supplementary material for: Morpho-Physiological and Transcriptome Changes in Tomato Anthers of Different Developmental Stages under Drought Stress
Source: Cells. 2021 Jul 17;10(7):1809. doi: 10.3390/cells10071809 (PMC8305550; doi:10.3390/cells10071809)
Supplement: Supplementary file 1 [file cells-10-01809-s001.zip › cells-1291549-supplementary/Suplementary Files/Supplementary Tables/Tables S2, S3 and S4.pdf]

Table S2. Summary of the number of floral buds used in the analysis of flowering phenology, floral bud abortion, opened flower abortion and fruit set.

	No. of buds	No. of aborted buds	No. of opened flowers	No. of opened flowers that aborted	No. of opened flowers that set fruits
WW	521	0	521	118	403
DS	492	122	370	190	180
Total	1013	122	891	308	583

Values are totals of 3 biological replicates each with 4 plants, 6 trusses/plant. WW, well-watered; DS, drought stressed.

Table S3. Correlations between replications of gene expression levels.

Developmental stage	Treatment	Rep.1 vs. Rep. 2	Rep.1 vs. Rep. 3	Rep.2 vs. Rep. 3
PMC-MEI (≤2.8mm)	WW	0.99	0.99	0.99
TED-VUM (≥4mm <6.5mm)	WW	0.98	0.95	0.96
(BIN-MP (>7.0mm)	WW	0.97	0.93	0.95
PMC-MEI (≤2.5mm)	DS	0.99	0.98	0.98
TED-VUM (≥4mm <6.5mm)	DS	0.91	0.97	0.86
BIN-MP (>7.0mm)	DS	0.97	0.97	0.97

WW, Well-watered; DS, Drought-stressed; PMC- MEI, anthers at pollen mother cell to meiotic stage; TED-VUM, anther at tetrad to uninucleate microspore stages; BIN-MP, anther a binucleate to mature pollen stage.

Table S4. Summary of reads and mapping information.

Sample name	Total	Average	Percentage
Total reads	924,197,894	51,344,327	
Total mapped	878,030,710	48,779,484	95.0
Multiple mapped	15,951,975	886,221	1.8
Uniquely mapped	862,078,735	47,893,263	98.2
Reads map to '+'	430,424,146	23,912,453	50
Reads map to '-'	431,654,589	23,980,811	50

Values are averages of 3 independent biological replicates.
